# Role of miRNAs in Epicardial Adipose Tissue in CAD Patients with T2DM

**DOI:** 10.1155/2016/1629236

**Published:** 2016-08-14

**Authors:** Yang Liu, Wenbo Fu, Mu Lu, Shitao Huai, Yaqin Song, Yutao Wei

**Affiliations:** ^1^Medicine Department, Shihezi University, Shihezi, Xinjiang 832000, China; ^2^Department of Cardiology, People's Hospital of Xinjiang Uygur Autonomous Region, Urumqi, Xinjiang 830000, China; ^3^Department of Thoracic and Cardiovascular Surgery, The First Affiliated Hospital of Shihezi University School of Medicine, Shihezi, Xinjiang 832008, China

## Abstract

*Background*. Epicardial adipose tissue (EAT) is identified as an atypical fat depot surrounding the heart with a putative role in the involvement of metabolic disorders, including obesity, type-2 diabetes mellitus, and atherosclerosis. We profiled miRNAs in EAT of metabolic patients with coronary artery disease (CAD) and type-2 diabetes mellitus (T2DM) versus metabolically healthy patients by microarray. Compared to metabolically healthy patients, we identified forty-two miRNAs that are differentially expressed in patients with CAD and T2DM from Xinjiang, China. Eleven miRNAs were selected as potential novel miRNAs according to* P* value and fold change. Then the potential novel miRNAs targeted genes were predicted via TargetScan, PicTar, and miRTarbase, and the function of the target genes was predicted via Gene Ontology (GO) analysis while the enriched KEGG pathway analyses of the miRNAs targeted genes were performed by bioinformatics software DAVID. Then protein-protein interaction networks of the targeted gene were conducted by online software STRING. Finally, using microarray, bioinformatics approaches revealed the possible molecular mechanisms pathogenesis of CAD and T2DM. A total of 11 differentially expressed miRNAs were identified and among them, hsa-miR-4687-3p drew specific attention. Bioinformatics analysis revealed that insulin signaling pathway is the central way involved in the progression of metabolic disorders.* Conclusions*. The current findings support the fact that miRNAs are involved in the pathogenesis of metabolic disorders in EAT of CAD patients with T2DM, and validation of the results of these miRNAs by independent and prospective study is certainly warranted.

## 1. Introduction

Coronary artery disease (CAD) remains one of the most common causes of morbidity and mortality in diabetic patients [1]. Thus improving the understanding of the etiology associated with CAD is highly important. Epicardial adipose tissue (EAT) is suggested to play an important role in the progression of metabolic syndrome [2]. Several findings implicate that EAT thickness may be a useful indicator for T2DM and obesity [2, 3]. Studies have shown that EAT generates several bioactive molecules, such as anti- and proinflammatory mediators and cytokines [4], which may significantly enhance paracrine effects on cardiac function or produce a variety of effects that affect many physiological processes [5]. Nowadays, beside the main factors including obesity, hypertension, and dyslipidemia, novel risk factors such as chronic low-grade inflammation, oxidative stress, and endothelial dysfunction are accepted as the decisive factors to highlight this increased cardiovascular risk in human beings [6, 7]. Experimental and clinical studies have suggested that EAT may cause CAD [8]. EAT, visceral fat depot of the heart, was found to be associated with CAD, T2DM, and other metabolic disorders [6, 9, 10]. EATs are metabolically active visceral fat deposits found around the heart, between the pericardium and myocardium [11], which are strongly associated with cardiovascular diseases (CVD) including CAD and the development of cardiac arrhythmias, predominantly due to the secretion of bioactive mediators and cytokines [12]. T2DM plays a key role in the development of CVD. EAT has great interplay with diabetic patients and has potential to influence CVD. Owing to its close proximity to the heart and coronary vasculature, EAT exerts a direct metabolic impact by secreting free fatty acids and proinflammatory factors and decreased anti-inflammatory adipokines, which promote CVD locally [9]. MicroRNAs (miRNAs) are a class of about 21–25 nucleotides in length and small noncoding RNA molecules with essential roles, of which any alteration leads to several conditions. They were first identified in 1993, and the term microRNA was created in 2001 [13]. The main functions of miRNAs are to downregulate the target gene expression in translational repression and cleavage of mRNA and in a wide range of biological processes [13]. Decisive regulatory functions exhibited by the miRNA are associated with various human diseases such as human cancer and heart disease. In addition to the link with cancer, microRNAs play a vital role in the control of cardiac-related diseases. For multiple forms of heart disease, including ventricular wall, maintenance of cardiac rhythm myocyte growth, and contractility, the misexpressions of miRNAs were shown to be necessary. However, in the literature, data regarding the relationship between miRNAs and EAT in CAD patients T2DM is scant. Hence, we aimed to investigate the role of miRNAs in EAT. We also sought to predict the targets of novel miRNAs in EAT in patients compared with the results that are obtained from control subjects [6]. The current study was carried out to assess the changes in the EAT levels of miRNAs in subjects suffering from T2DM and CAD compared to healthy control ones. In addition, bioinformatics analyses were carried out in order to find out how these possible miRNAs are associated with the incidence and pathogenesis of CAD on top of T2DM. In the present study, we depicted comparative miRNAs in “metabolically healthy” patients without metabolic disorders and in metabolic patients with CAD and T2DM. This strategy allowed us to identify a set of miRNAs characterizing EAT in health and disease as well as potential novel biological processes characterizing EAT in CAD patients with T2DM.

## 2. Methods

### 2.1. Subjects

EAT samples were taken from 10 subjects of both genders, in the Department of Thoracic and Cardiovascular Surgery, the First Affiliated Hospital of Shihezi University School of Medicine, in which levels of miRNAs expression in EAT (using microarray) and routine parameters were measured. Subjects were divided into two groups, 5 in each group as follows: patients with T2DM and CAD and metabolically healthy control subjects without T2DM and CAD. Ten Asian patients were recruited, 5 underwent cardiac valve surgery (no evidences of CAD/T2DM/carotid atherosclerosis/metabolic syndrome), and 5 underwent coronary artery bypass graft surgery (CAD + T2DM group). The study protocol was approved by the Medical Ethics Committee of Shihezi University (School of Medicine, Xinjiang, China). Written informed consent was obtained from all subjects included in the study. This was a cross-sectional study and a review of medical records (including information on sex, age, height, weight, medications, disease duration, smoking, and history of other diseases) was undertaken. Control subjects were chosen from metabolically healthy individuals according to NCEP ATP III Metabolic syndrome criteria (two or less metabolic criteria; TG ≥ 1.7 mmol/L, blood pressure ≥ 130/85 mmHg, glucose ≥ 5.6 mmol/L, HDL-C: men < 1.03 mmol/L and women < 1.30 mmol/L, and waist circumference: men < 102 cm and women < 88 cm) as previous said [6, 14]. miRNAs microarray expression analyses were conducted on RNA extracted from perivascular EAT using the Human-MicroRNA Expression Kits.

### 2.2. RNA Extraction and Purification

Total RNA, including the miRNAs, was extracted from the EAT and purified using* mir*Vana*™* miRNA Isolation Kit (Cat. number AM1561, Ambion, Austin, TX, US), following the manufacturer's instructions, and checked for a RNA integrity number (RIN) to inspect RNA integration by an Agilent Bioanalyzer 2100 (Agilent Technologies, Santa Clara, CA, US). RIN ≥ 6.0 and 28S/18S ≥ 0.7 were used for the miRNA array analysis.

### 2.3. RNA Labeling

miRNA molecular in total RNA was labeled by miRNA Complete Labeling and Hyb Kit (Cat. number 5190-0456, Agilent Technologies, Santa Clara, CA, US) following the manufacturer's instructions, labeling section.

### 2.4. Array Hybridization

MiRNA microarray assays were performed using the Agilent Human miRNA (8 *∗* 60 K) V21.0 microarray platform (design ID: 70156) at Shanghai Biotechnology Co., Ltd. (Shanghai, China). Each slide was hybridized with 100 ng Cy3-labeled RNA using miRNA Complete Labeling and Hyb Kit (Cat. number 5190-0456, Agilent Technologies, Santa Clara, CA, US) in hybridization oven (Cat. number G2545A, Agilent Technologies, Santa Clara, CA, US) at 55°C, 20 rpm for 20 hours according to the manufacturer's instructions, hybridization section. After hybridization, slides were washed in staining dishes (Cat. number 121, Thermo Shandon, Waltham, MA, US) with Gene Expression Wash Buffer Kit (Cat. number 5188-5327, Agilent Technologies, Santa Clara, CA, US).

### 2.5. Data Acquisition

Slides were scanned by Agilent Microarray Scanner (Cat. number G2565CA, Agilent Technologies, Santa Clara, CA, US) and Feature Extraction software 10.7 (Agilent Technologies, Santa Clara, CA, US) with default settings. Raw data were normalized by Quantile algorithm, Gene Spring Software 12.6 (Agilent Technologies, Santa Clara, CA, US).

### 2.6. Differential miRNAs Targeted Gene Prediction

The differential miRNAs targets predicted by computer-aided algorithms were obtained from TargetScan, PicTar, and miRBase targets [15]. More detailed information can be acquired from online software (http://pictar.mdc-berlin.de/cgi-bin/new_PicTar_vertebrate.cgi; http://microrna.sanger.ac.uk/cgi-bin/targets/v5/search.pl; http://www.targetscan.org/).

### 2.7. The Interaction Network and Signaling Pathway Analysis of Differential microRNA and mRNA

DAVID [16–18], a bioinformatics analysis software, is used for the analysis of the enriched KEGG (Kyoto Encyclopedia of Genes and Genomes) signaling pathway analysis for the interactions between microRNAs and mRNAs (http://david.abcc.ncifcrf.gov/). Online software Gene Ontology (http://geneontology.org/) was employed to perform GO enrichment analysis [19, 20]. The microRNA and mRNA of differential expression in patients were uploaded to DAVID and Gene Ontology for analysis.

### 2.8. Protein-Protein Interactions (PPI) Network Analysis

A number of abnormal mRNAs were found in the interaction analysis between miRNAs and mRNA. Therefore, to further understand the function of microRNA in the network, the PPI analysis was performed in the protein products of mRNAs to find out the key proteins. The selected targeted genes were put into the STRING (Search Tool for the Retrieval of Interacting Genes) database (http://string-db.org/), a metaresource that collects most of the available information on protein-protein associations and scores and weights it and augments it with predicted interactions and with the results of automatic opuses-mining searches to match the interactions of proteins [21, 22].

### 2.9. Statistical Analysis

Descriptive statistics for each variable were determined. Results for continuous variables were demonstrated as mean ± standard deviation. Statistical significant difference between the groups was determined by the chi-square test for categorical variables and unpaired Student's *t*-test for continuous variables. Differentially expressed targeted genes were studied using bioinformatics analysis and statistical analysis allowed algorithm of the selected software.

## 3. Results

### 3.1. Baseline Characteristics of Patients

The baseline characteristics of 5 patients and 5 control subjects were shown in Table 1. There were no differences with respect to the following variables between patients and control subjects, age, gender, waist circumference (WC), systolic blood pressure (SBP), diastolic blood pressure (DBP), and body mass index (BMI). Compared to “controls,” CAD patients with T2DM were characterized by significantly increased waist-hip ratio, LDL-C, and systemic inflammation, while HDL-C was decreased.

### 3.2. Data of Microarray

**Figure 1:**
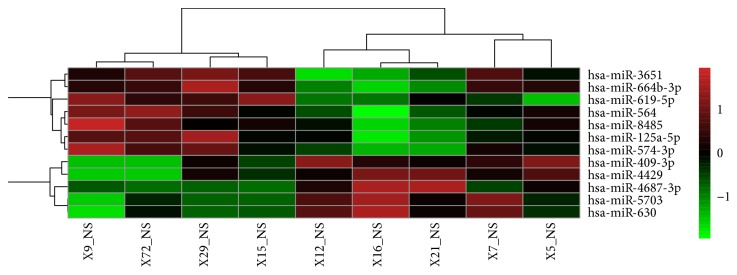
Unsupervised hierarchical clustering (heat map). Heat map generated by hierarchical clustering for differentially expressed miRNAs in the EAT from CAD with T2DM patients versus control subjects. Hierarchical clustering for differentially expressed miRNAs in CAD with T2DM (*n* = 4) versus control (*n* = 5) (*P* < 0.05 and fold change > 2 times). Columns display the clustering of EAT samples; rows show the clustering of genes. The expression intensity of each miRNA in each sample varies from red to green, which indicates relative high or low expression, respectively. Expression clusters representing different patterns of upregulation to downregulation are depicted on the side of figure.

In order to measure the miRNAs expression patterns that characterize EAT in CAD patients with T2DM from EAT in control group, we used the whole-genome miRNAs microarrays. Overall, the resulting signal intensity of miRNAs genes is statistically different at the Wilcoxon signed-rank test in EAT in both groups, thus underscoring the profound diversity of EAT. Unsupervised hierarchical clustering was presented in Figure 1. Compared to metabolically healthy patients, we identified forty-two miRNAs that are differentially expressed in patients with CAD and T2DM (26 downregulated; 16 upregulated, data was not shown). Eleven miRNAs were selected as potential novel miRNAs according to *P* value and fold change (Table 2: 6 were significantly downregulated; 5 were significantly upregulated in both subgroups of patients with *P* < 0.05; fold change > 2 times).

### 3.3. Results of Bioinformatics Analyses

To depict the possible role of miRNAs in EAT, we selected these miRNAs as potential novel biomarkers that were significantly different (patients versus controls) in the overall population (*P* < 0.05; fold change > 2). To provide a framework for interpretation of our results, we then functionally clustered significant biological pathways using the bioinformatics analyses.

#### 3.3.1. Bioinformatics Analyses of miRNAs Targeted Genes

**Figure 2:**
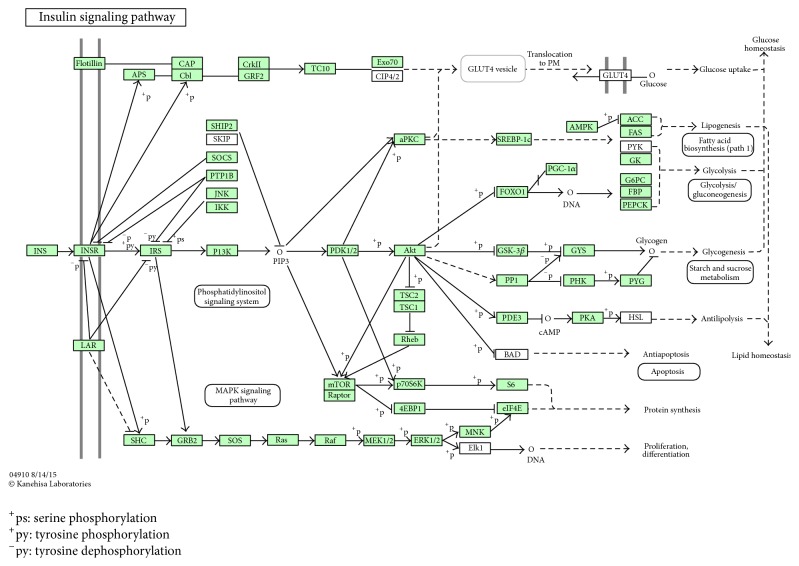
Insulin signaling pathway. ERK1/2(MAPK1), GRB2, PKA (PRKAR), P13K (PIK3), and PDK1/2 (PDPK1) are the main target genes of differential miRNAs.

In order to investigate the possible regulation mechanisms of miRNAs in the process of CAD complicated with T2DM, we first utilized three online bioinformatics databases (TargetScan, PicTar, and miRBase targets) to select plausible targets and validated targets of miRNAs and finally obtained target genes for the following analysis; then we analyzed biological processes, molecular functions, and cellular components through Gene Ontology and enriched KEGG pathways by DAVID. The results showed that the predicted target genes mainly are enriched in the following biological processes: detection of chemical stimulus involved in sensory perception of smell, G-protein coupled receptor signaling pathway, translation, axon guidance, protein phosphorylation, and so forth (*P* < 0.001), they mainly are enriched in the following molecular functions: G-protein coupled receptor activity, olfactory receptor activity, protein binding, structural constituent of ribosome, and so forth (*P* < 0.001), and significant cellular components were extracellular region and Golgi apparatus (*P* < 0.001). Enriched KEGG pathways by DAVID displayed in KEGG pathway database showed that the predicted target genes of miRNAs were significantly enriched in the insulin signaling pathway (Figure 2), adipocytokine signaling pathway, MAPK signaling pathway, FoxO signaling pathway, and other signaling pathways (*P* < 0.05).

#### 3.3.2. Protein-Protein Interaction (PPI) Network

**Figure 3:**
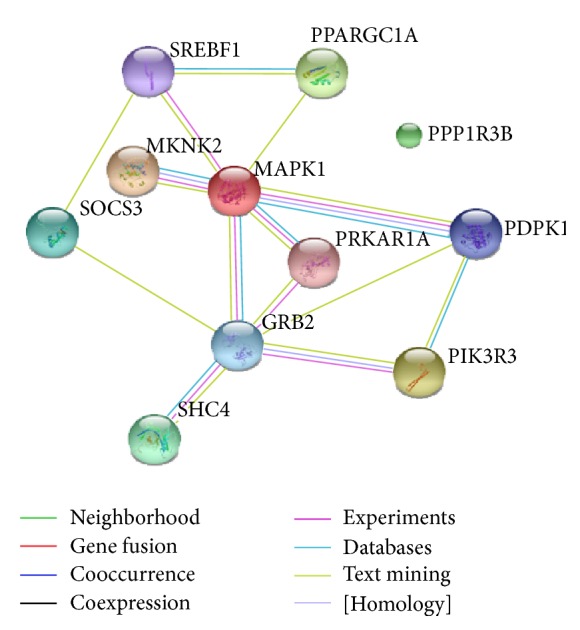
Protein-protein interaction (PPI) network of target proteins of the selecting miRNAs.

We analyzed the protein-protein interaction network of selecting miRNAs target genes using STRING 10 and removed the target genes which were linked to lax isolated nodes through data analysis; the result showed that the interaction existed in total 148 proteins targeted by the predicted genes, which together formed the target gene interaction network. The network consists of 148 nodes which represent 148 proteins and many lines with different colors that represent the types of evidence for the association. From the result we can see that PDPK1, PIK3R3, PPP1R3B, PRKAR1A, SOCS3, SREBF1, PPARGC1A, SHC4, MAPK1, GRB2, and MKNK2 played key roles in maintaining stability in the network, especially PIK3R3, MAPK1, and GRB2 whose connections were very close, so the protein encoded by them may be important downstream target proteins (Figure 3).

## 4. Discussion

Better understanding of the biological characteristics can provide vital theoretical basis for the prevention and treatment of disease. The aim of this study was to investigate the profiles of miRNAs and the interaction network of novel microRNA and mRNA as well as related signaling pathway in EAT through analyzing the expression profile of microRNA and mRNA to provide novel insights in the biological characteristics in CAD patients with T2DM. According to newly added studies, it is clear that miRNAs are involved in the regulation of metabolic and inflammation functions, and the alterations in CAD and T2DM diseases have been analyzed through the identification and evaluation of miRNAs profiles in patients as in animal models [13, 23, 24]. microRNAs are key components of many cellular processes. Different studies have demonstrated that miRNA expression is tissue-specific, tightly regulated during embryogenesis, and overexpressed/underexpressed in many diseases, including CAD and T2DM pathologies [25]. They are easily detected in a quantitative way by real-time polymerase chain reaction (qRT-PCR) or microarrays and by other less frequently used identification methods, such as PCR-based restriction fragment length polymorphisms (PCR-RLFP), traditional northern blotting, direct sequencing using next generation sequencing (NGS), and platforms ligation based measurement [26]. Risk prediction for T2DM and CVD remains suboptimal even after the introduction of global risk assessment by various methods. This has prompted the search for additional biomarkers [27]. EAT is a source of several inflammatory mediators in high-risk cardiac patients [4]. Adipose tissue may function as an endocrine organ that contributes to an inflammatory burden in patients at risk of CVD. Obesity, adiposopathy, and insulin resistance induce EAT enlargement, inflammation, and dysfunction and trigger CAD [10]. EAT is an atypical fat depot surrounding the heart with a putative role in the development of atherosclerosis [28]. Given the close anatomic relationship between perivascular EAT and coronary arteries and the positive correlation between EAT and the presence of coronary atherosclerosis, several results point to EAT as a putative actor in CAD and/or T2DM [3, 10]. EAT thickness is an independent risk factor for CAD. EAT could thus be able to modulate heart and coronary artery pathophysiology, and mounting evidences point to EAT as a candidate player in pathophysiology of CAD. miRNAs act on multiple targets and complex pathogenesis, thus representing candidate regulators of adipocyte differentiation, metabolic homeostasis, and inflammation. miRNAs have also been described as differentially modulated in adipose tissue during metabolic disease, thus being considered candidate biomarkers for metabolic disorders, CAD, T2DM, and putative targets for therapy [28]. We present evidence that a profile of miRNAs was dysregulated in EAT in CAD patients with T2DM compared to metabolic health patients, supporting the concept that miRNAs in EAT are involved in pathogenesis of CAD and/or T2DM. The current study identified a total of 11 differentially expressed miRNAs, and among them, hsa-miR-4687-3p drew specific attention for the largest targeted genes which were identified. Particularly, bioinformatics analysis revealed that insulin signaling pathway is the central way involved in the progression of metabolic disorders. Previous studies indicated that the circulating levels of miR-133a can be used as a predictor for diagnosing CAD, since increased miR-133a level may be used to predict both the presence and severity of coronary lesions in CAD patients [29]. Evidence from a recent study had showed that miR-370 was significantly increased in patients with T2DM and CAD and CAD only and patients with T2DM [23]. This was also concomitant with another study that showed the use of certain miRNA imprints including miR-370, in the screening of patients at risk for developing CAD [30]. Because of the highly metabolic paracrine and endocrine functions of EAT, it has been proposed to play a role in the pathogenesis of CVD by releasing proinflammatory and proatherogenic factors. EAT plays important roles in CAD, not only in its location, but also by its blood supply. EAT derives its blood supply from coronary circulation. There is a functional and anatomic relationship between EAT and muscular components of the heart as these components share the same coronary blood supply. The release of proinflammatory and proatherogenic factors into the circulation advancing CVD is more significantly linked to the progression of CAD [10]. It is the close anatomical relationship between EAT and the coronary arteries combined with its biologically active properties that participates in the pathogenesis of diabetic coronary atherosclerosis [31]. EAT-specific miRNAs are miR-196b-5p, miR-196a-5p (a promoter of brown adipogenesis), miR-18a-3p (a member of the miR-17/92 cluster that promotes adipocyte differentiation), and miR-10a-3p (an anti-inflammatory agent) which were analyzed by one article that also supported our study. In EAT of CAD patients, miR-135b-3p (a direct target of inflammatory pathways) was found upregulated, while miR-455-3p (a driver of during brown adipocyte differentiation), miR-193b-3p (promoting adiponectin secretion in human adipocytes), and Let-7a-3p and miR-127-3p (negative modulators of inflammatory pathways) were found downregulated [32, 33]. These findings are in accordance with our results that miRNAs are involved in the pathogenesis of metabolic disorders in EAT of CAD patients with T2DM. Overall, we were able to depict a novel miRNA signature of EAT in CAD patients with T2DM characterized by dysregulated miRNAs profile which are probably involved in pathogenesis of CAD. The results of the current study support the hypothesis that miRNA expression is deregulated in epicardial adipose tissue in patients suffering from T2DM and CAD disease.

We provided a comprehensive potential novel miRNAs expression signature of EAT in CAD patients with T2DM, and we showed that the targets of the miRNAs are necessarily associated with the pathogenesis of CAD and T2DM.

## Figures and Tables

**Table 1:** Baseline characteristics of patients.

	Non-CAD + T2DM (*n* = 5)	CAD + T2DM (*n* = 5)	*P* value
Age (years)	61.8 ± 5.2	54.6 ± 7.0	0.392
BMI (kg/m^2^)	29.47 ± 5.83	28.63 ± 4.26	0.213
WC (cm)	99.64 ± 10.67	101.23 ± 10.34	0.492
Waist-hip ratio	0.96 ± 0.14	0.99 ± 0.09	<0.001
FPG (mg/dL)	106.5 (98.5–115.5)	116.0 (100.5–138.0)	0.103
Total cholesterol	166.48 ± 37.10	159.55 ± 39.41	0.335
LDL-C (mg/dL)	93.51 ± 26.59	98.44 ± 23.14	<0.01
HDL-C (mg/dL)	40.66 ± 10.08	34.39 ± 9.64	<0.01
Triglyceride (mg/dL)	139 (105.5–223.5)	154 (106.0–234.0)	0.276
hsCRP (mg/dL)	1.03 ± 2.31	3.15 ± 5.22	<0.001
Adiponectin (*μ*g/mL)	12.37 ± 6.55	9.16 ± 4.78	<0.05
Fibrinogen (mg/dL)	523.0 (453.0–638.0)	603.5 (510.5–766.5)	<0.01

FPG: fasting plasma glucose, LDL-C: low density lipoprotein cholesterol, HDL-C: high density lipoprotein cholesterol, and hsCRP: hypersensitive C reactive protein. Adiponectin, hsCRP, and fibrinogen indicate systemic inflammation.

**Table 2:** Disregulated miRNAs (CAD + T2DM versus control).

miRNA	*P* value	Fold change
hsa-miR-4429	0.04437405	0.16922769
hsa-miR-409-3p	0.012379586	0.184890055
hsa-miR-6802-5p	0.047496135	0.305662149
hsa-miR-5703	0.008804377	0.40973895
hsa-miR-630	0.020813173	0.43854972
hsa-miR-4687-3p	0.034377945	0.44430727
hsa-miR-3651	0.04317499	2.063333894
hsa-miR-574-3p	0.025198143	2.156938357
hsa-miR-619-5p	0.00179165	2.179715733
hsa-miR-664b-3p	0.04815202	2.325766896
hsa-miR-146b-5p	0.044983658	2.450112684

Lists of the deregulated miRNAs between CAD + T2DM and control. Fold change < 0.5 indicated downregulation significantly and fold change > 2 indicated upregulation significantly.
